# Familial longevity is associated with lower baseline bone turnover but not differences in bone turnover in response to rhTSH

**DOI:** 10.18632/aging.203511

**Published:** 2021-09-07

**Authors:** Ana Zutinic, Ferdinand Roelfsema, Hanno Pijl, Bart E. Ballieux, Rudi G.J. Westendorp, Gerard J. Blauw, Diana van Heemst

**Affiliations:** 1 Department of Internal Medicine, Division of Gerontology and Geriatrics, Leiden University Medical Center, Leiden, The Netherlands; 2 Department of Internal Medicine, Division of Endocrinology and Metabolic Diseases, Leiden University Medical Center, Leiden, The Netherlands; 3 Department of Clinical Chemistry and Laboratory Medicine, Leiden University Medical Center, Leiden, The Netherlands; 4 Public Health and Centre for Healthy Aging, University of Copenhagen, Copenhagen, Denmark

**Keywords:** longevity, thyroid, bone metabolism, rhTSH

## Abstract

Context: Offspring from long-lived families have a different thyroid status than controls, characterised by higher circulating levels of thyroid stimulating hormone (TSH) and similar levels of thyroid hormone. Expression of the TSH receptor has previously been observed on various extrathyroidal tissues, including bone. However, potential physiological consequences of differences in circulating TSH as observed in familial longevity on bone tissue remain unclear.

Objective: Based on the hypothesis that TSH may inhibit bone resorption, we explored whether offspring of long-lived families have lower bone turnover than controls at baseline as well as following a challenge with recombinant human TSH (rhTSH).

Methods: Bone turnover markers CTX and P1NP were measured in fasted morning samples from 14 offspring and 12 controls at baseline and at 24 hour intervals following 0.1 mg rhTSH i.m. administration for four consecutive days.

Results: At baseline, mean (SEM) CTX was 0.32 (0.03) ng/ml in offspring and 0.50 (0.04) ng/ml in controls, *p* < 0.01, whereas mean (SEM) P1NP was 39.6 (3.2) ng/ml in offspring and 61.8 (6.6) ng/ml in controls, *p* < 0.01. Following rhTSH administration, both CTX and P1NP levels transiently increased over time and normalized towards baseline after 72 h (general linear modelling: CTX time *p* = 0.01, P1NP time *p* < 0.01); the response was similar between offspring and controls.

Conclusions: Bone turnover markers were lower at baseline in offspring from long-lived families than in controls but increased similarly following an rhTSH challenge.

## INTRODUCTION

Even subtle differences within the euthyroid range have been associated with exceptional human longevity and with differences in survival at old age [1–3]. Although the precise underlying mechanism of this observation is unknown, it could be due to the role that the hypothalamus-pituitary-thyroid axis plays in influencing numerous physiological processes and tissues across the different stages of the lifespan, such as energy metabolism and tissue maintenance and repair [4, 5].

In the Leiden Longevity Study (LLS) [6, 7], we have recently shown that offspring from long-lived families (offspring) have a different thyroid status than similarly-aged controls [8]. The offspring had consistently higher circulating thyroid stimulating hormone (TSH) concentrations, as measured every 10 minutes over a 24 hour period, in the absence of differences in thyroid hormone levels or TSH bioactivity [8]. To assess potential mechanisms that may cause this difference, we consequently performed two thyroid challenge studies in a subgroup of the same cohort: one challenge with recombinant human TSH (rhTSH) [9] and another with thyroid hormone triiodothyronine (T3) [10]. We found that offspring had a lower thyroidal responsivity to rhTSH stimulation than controls [9] and similar thyroid hormone T3 turnover [10].

The physiological consequences of this lower thyroidal responsivity to TSH as well as the higher circulating TSH levels in absence of differences in thyroid hormones in offspring, and how and whether these contribute to their predisposition for longevity, remain unknown. In addition to an established role in energy metabolism, it has been suggested that thyroid hormones play crucial roles in tissue maintenance and repair, which are of key importance to longevity [11]. Interestingly, expression of the TSH receptor has been observed on various extrathyroidal tissues, including the pituitary gland, thymus, testes, kidney, liver, adipose tissue, and bone. Based on these observations, it has been suggested that in addition to thyroid hormone, TSH may have a role in other tissues that the thyroid gland, including bone tissue [12–14].

Although tissue maintenance and repair are generally notoriously difficult to measure in humans [15], bone tissue turnover can be assessed reliably [16] through measurement of the serum osteoclastic bone resorption marker collagen type 1 C-terminal cross-linked telopeptide (CTX) and the osteoblastic bone formation marker N-terminal propeptide of type 1 collagen (P1NP).

In the case of bone, based on earlier studies, it was suggested that TSH inhibits osteoclastic bone resorption and that in addition to higher levels of thyroid hormone, low circulating TSH may contribute to bone loss [17–19]. Based on these observations, the hypothesis was formulated that the net result of slightly higher circulating TSH (while circulating levels of thyroid hormone are comparable), as observed in familial longevity, might be a slightly lower rate of bone resorption, which might protect against age-associated bone loss, and thus contribute to healthy longevity. The aim of the present study was to compare the levels of the bone markers CTX and P1NP in offspring from long-lived families and controls at baseline and following a rhTSH challenge over a four consecutive day study period. The specific objectives were threefold: i) to compare baseline levels of bone resorption marker CTX and bone formation marker P1NP between offspring and partners, ii) to explore whether circulating levels of these markers of bone resorption and bone formation would change after stimulation with rhTSH, and iii) to explore potential differences between offspring and partners in the response of these markers of bone resorption and bone formation after stimulation with rhTSH.

## RESULTS

### Inclusions

**Figure 1:**
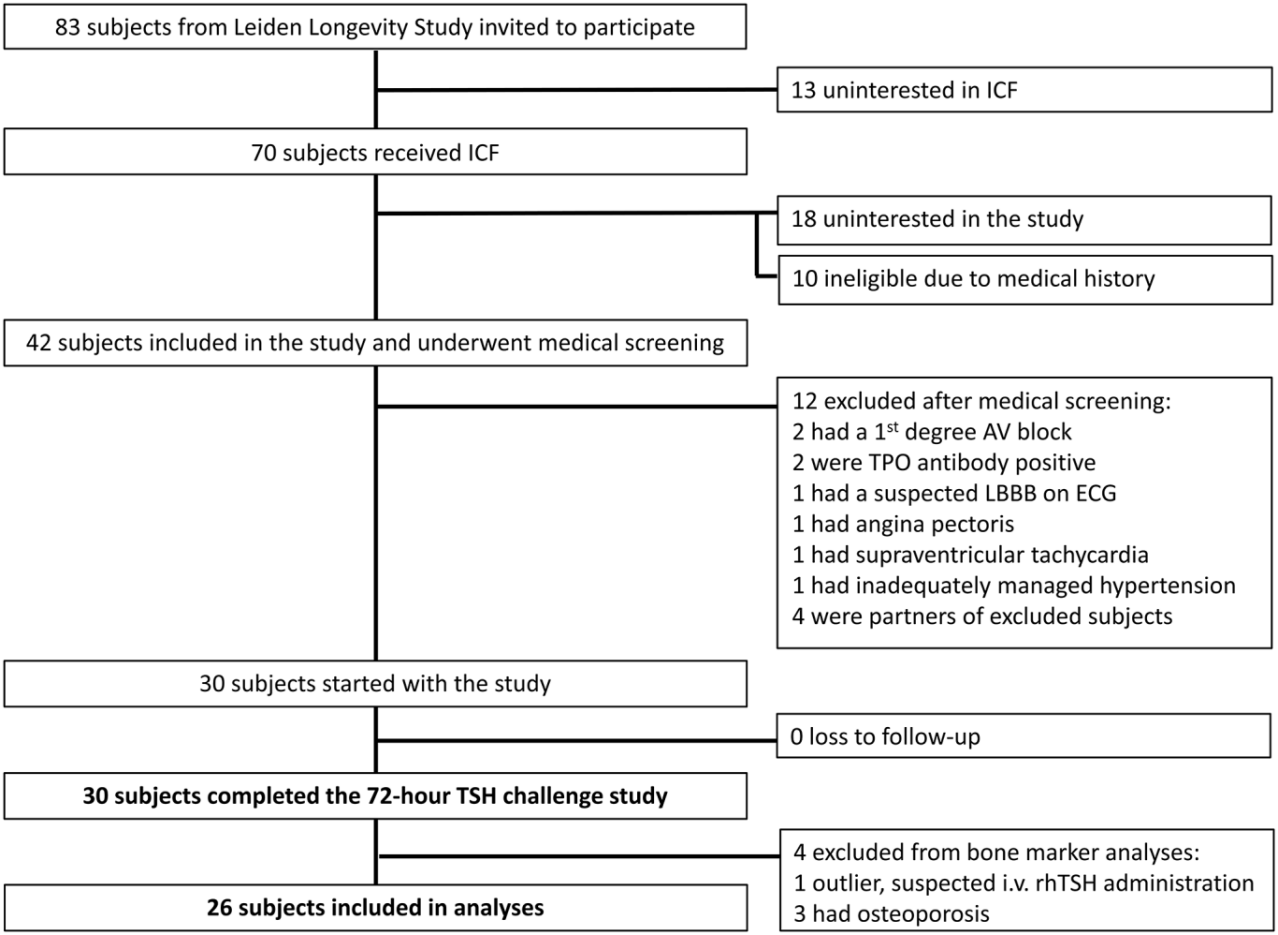
**Inclusion flow chart bone marker analysis in the recombinant human thyroid stimulating hormone (rhTSH) study.** Abbreviations: ICF: informed consent form; AV: atrioventricular; TPO: thyroid peroxidase; LBBB: left bundle branch block; ECG: electrocardiogram; i.v.: intravenous.

The inclusion flow chart of subjects for the study is presented in Figure 1. In total, 30 subjects from the Leiden Longevity Study participated in the rhTSH study. One subject (male offspring) was excluded from bone marker analysis due to suspected i.v. rhTSH administration. Three subjects (female controls) were excluded from bone marker analysis due to osteoporosis. Bone marker analysis was performed in 26 subjects (14 offspring and 12 controls).

### Group characteristics

Baseline characteristics of the study population are presented in Table 1. Offspring and controls comprised an active, healthy, high middle-aged population and the groups were similar regarding age, sex, BMI and exercise level. Maternal age and paternal age were higher in offspring compared to controls (*p* < 0.01 and *p* = 0.03, respectively), in accordance with the longevity phenotype selection criteria. The distribution of males and females was similar between the offspring and control group (*p* = 0.72). Laboratory measurements of thyroid hormones and liver function were all within normal range and similar between offspring and controls, with baseline TSH being significantly higher in offspring than in partners (*p* < 0.04), a distinction characterising this cohort. Both groups had a mean kidney function within normal range (GFR >60 mL/min per 1.73 m^2^), which was similar between offspring and partners (*p* = 0.11). One participant, a female offspring, had GFR 54 at screening, but was included in the study due to absence of any indication of chronic (kidney) disease.

**Table 1:** Baseline characteristics of the study population.

	**Offspring (*n* = 14)**	**Controls (*n* = 12)**	***P* value**
**Demographics**			
Age mother years^*^	93 (91–97)	77 (69–90)	**<0.01**
Age father years^*^	93 (73–96)	78 (64–86)	**0.03**
Male *n (%)*	8 (57)	6 (50)	0.72
Age *years*	69 (5)	68 (7)	0.87
**Anthropometrics**			
BMI *kg/m^2^*	25.8 (4.3)	26.1 (3.3)	0.84
Height *cm*	173.7 (10.9)	173.6 (9.0)	0.97
Fat mass *kg*	23.9 (7.2)	25.2 (5.8)	0.62
Lean mass *kg*	54.1 (12.9)	53.0 (13.3)	0.83
**Vitamins and exercise**			
Vitamin D supplementation *n* (%)	1 (7)	1 (8)	0.27
Regular weekly exercise *n* (%)	12 (86)	9 (75)	0.49
- Low intensity (walking) *n* (%)	4 (29)	4 (33)	0.8
- High intensity (jogging, tennis) *n* (%)	8 (57)	5 (42)	0.44
**Laboratory measurements**			
GFR *ml/min per 1.73 m^2^*	71.2 (13.9)	78.9 (8.6)	0.11
AST *U/L*	22.3 (4.1)	25.3 (7.4)	0.2
ALT *U/L*	19.6 (5.3)	21.7 (5.0)	0.32
Baseline TSH *mU/L*	3.3 (1.7)	2.07 (1.0)	**0.04**
Baseline fT4^*^ *pmol/L*	13.9 (13.0–15.8)	15.3 (14.4–15.7)	0.11

All values are mean (standard deviation) unless otherwise stated. Abbreviations: BMI: body mass index; GFR: glomerular filtration rate; AST: aspartate transaminase; ALT: alanine transaminase; TSH: thyroid stimulating hormone; fT4: free T4; fat mass: fat mass, in kilograms; as measured by Bioelectrical Impedance Analysis; lean mass: fat free mass, in kilograms; as measured by Bioelectrical Impedance Analysis. ^*^Median (interquartile range).

### Baseline bone markers

**Figure 2:**
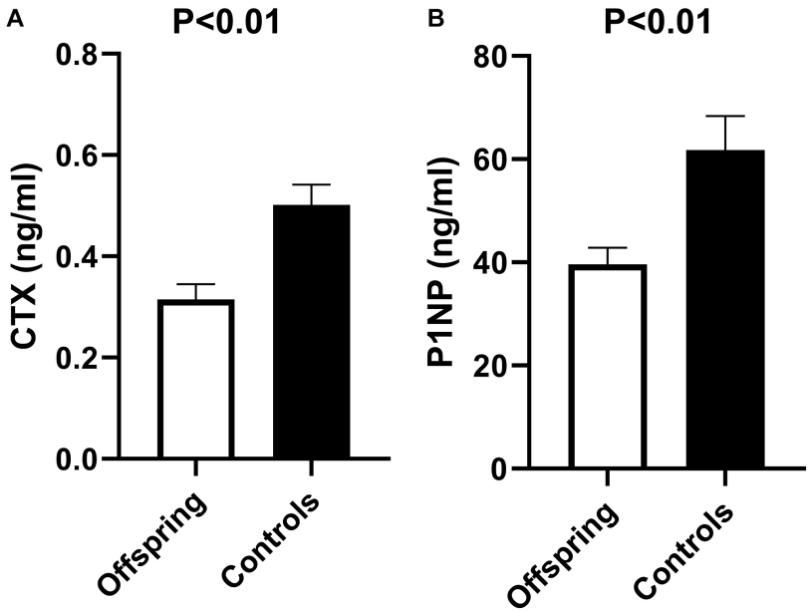
**Bone resorption (CTX) and bone formation (P1NP) markers in offspring versus controls at baseline of rhTSH study.** (**A**) Mean CTX at baseline of rhTSH study in offspring (*n* = 14) and controls (*n* = 12), *p* value <0.01. (**B**) Mean P1NP at baseline of rhTSH study in offspring (*n* = 14) and controls (*n* = 12), *p* value <0.01. Abbreviations: rhTSH: recombinant human thyroid stimulating hormone; CTX: collagen type 1 C-terminal cross-linked telopeptide (CTX); P1NP: N-terminal propeptide of type 1 collagen. Error bars: standard error of the mean. *P* value <0.05 was considered statistically significant.

Baseline bone markers in offspring and controls are shown in Figure 2. Bone resorption marker CTX was significantly lower in offspring than in controls (mean (SEM) 0.32 (0.03) ng/ml and 0.50 (0.04) ng/ml, respectively, *p* < 0.01). The same was observed for the bone formation marker P1NP, which was also significantly lower in offspring than in controls (mean (SEM) 39.6 (3.2) ng/ml and 61.8 (6.61) ng/ml, respectively, *p* < 0.01).

The bone markers were not different between men and women (CTX: mean (SEM) 0.36 (0.03) ng/ml and 0.45 (0.06) ng/ml respectively, *p* = 0.16; P1NP: mean (SEM) 42.8 (2.8) ng/ml and 58.2 (7.8) ng/ml respectively, *p* = 0.06). Both male and female offspring had lower bone markers than male and female controls. Within males, P1NP was significantly lower in offspring than in controls (mean (SEM) 36.7 (3.0) and 50.8 (3.0) ng/ml in offspring and controls respectively, *p* < 0.01), while CTX followed a similar trend (mean (SEM) 0.31 (0.04) and 0.42 (0.04) ng/ml in offspring and controls respectively, *p* = 0.10). Within females, CTX was lower in offspring than controls (mean (SEM) 0.32 (0.05) and 0.58 (0.08) ng/ml in offspring and controls respectively, *p* = 0.01), and P1NP followed the same trend (mean (SEM) 43.5 (6.6) and 72.8 (11.7) ng/ml in offspring and controls respectively, *p* = 0.05).

### Bone markers following rhTSH administration

**Figure 3:**
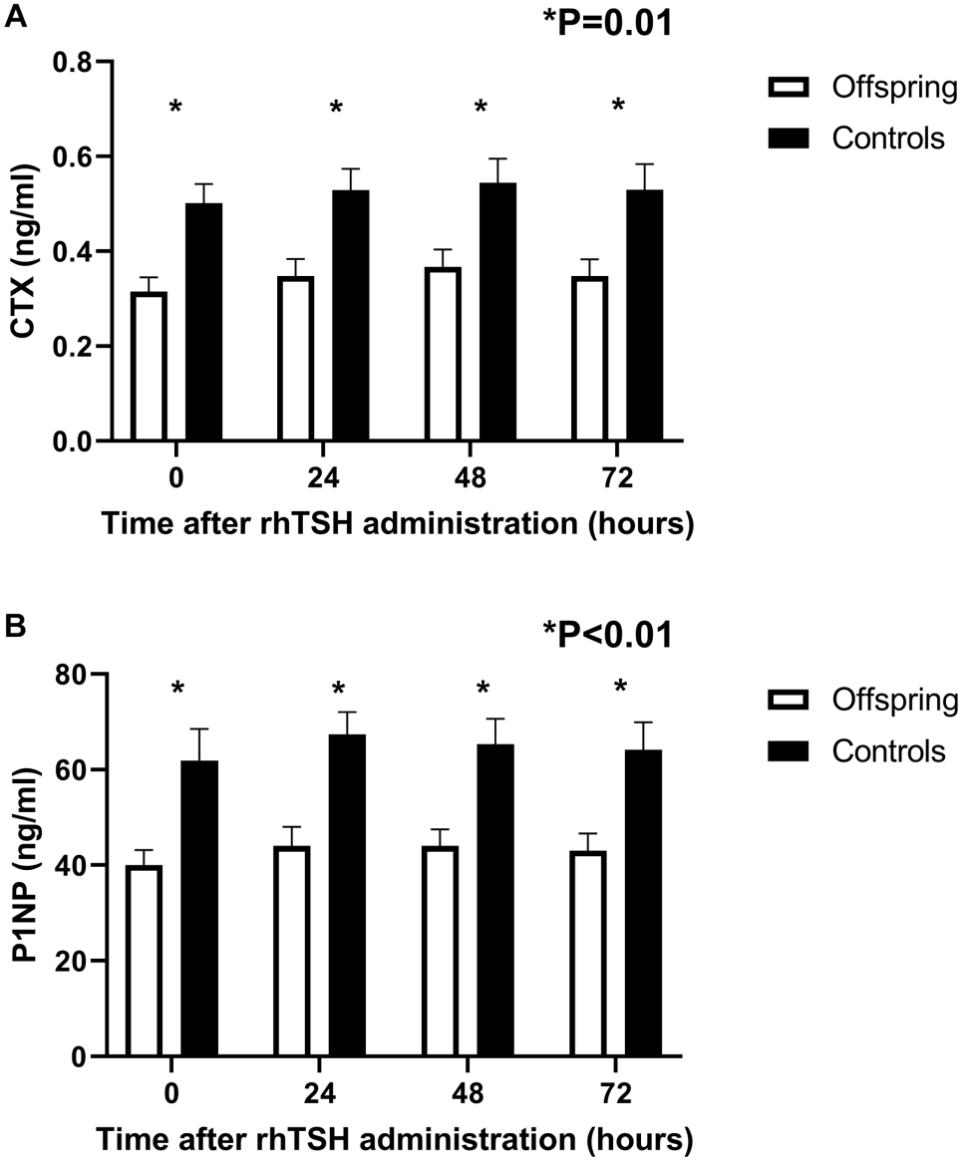
**Concentration profiles of bone resorption (CTX) and bone formation (P1NP) markers over time in offspring from long-lived families (*n* = 14) and controls (*n* = 12) following a challenge with 0.1 mg rhTSH.** (**A**) Mean circulating CTX at 24-hour intervals following rhTSH administration. (**B**) Mean circulating P1NP at 24-hour intervals following rhTSH administration. rhTSH: recombinant human thyroid stimulating hormone, CTX: collagen type 1 C-terminal cross-linked telopeptide (CTX), P1NP: N-terminal propeptide of type 1 collagen. Error bars: standard error of the mean.

The concentration profiles of bone markers CTX and P1NP over time following rhTSH administration are shown in Figure 3.

Upon visual inspection, the response in bone markers to rhTSH administration seemed similar in offspring and controls. Both CTX and P1NP markers increased following rhTSH administration (CTX ANOVA = 0.01, P1NP ANOVA <0.01, Table 2), with CTX displaying the highest increase 48 hours after administration while P1NP reached highest value at 24 hours following rhTSH administration. Bone markers normalized towards baseline at 72 hours following rhTSH administration.

**Table 2:** Assessment of effects of time, and its interaction with group and gender using general linear model analysis of bone resorption and formation markers following recombinant human TSH administration in 26 study subjects (14 offspring and 12 controls).

	**Bone resorption (CTX)**	**Bone formation (P1NP)**
**Within**		
** Time**	***p* = 0.01**	***p* < 0.01**
Time^*^group	*p* = 0.95	*p* = 0.85
Time^*^gender	*p* = 0.14	*p* = 0.95

Despite the increase in bone markers following rhTSH administration, concentration of both CTX and P1NP remained lower in offspring than in controls throughout the study (CTX: *p* = 0.01, 0.01 and 0.01 at 24, 48 and 72 hours, respectively; P1NP: *p* = <0.01, <0.01 and <0.01 at 24, 48 and 72 hours, respectively).

Testing for interaction using general linear modelling confirmed that the effect of rhTSH administration on bone markers was not statistically different between offspring and controls, nor between males and females (Table 2).

## DISCUSSION

Based on the hypothesis that TSH may inhibit bone resorption, the specific objectives of this study were i) to compare baseline levels of bone resorption marker CTX and bone formation marker P1NP between offspring and controls, ii) to explore whether circulating levels of these markers of bone resorption and bone formation would change after stimulation with rhTSH, and iii) to explore potential differences between offspring and controls in the response of these markers of bone resorption and bone formation after stimulation with rhTSH.

The novel findings of this study are threefold. Firstly, familial longevity is characterized by lower bone turnover at baseline, indicating that bone tissue is yet another organ system possibly impacted by the longevity phenotype. Secondly, rhTSH administration increases bone turnover markers, indicating that exogenous TSH and/or endogenous thyroid hormones are drivers of bone turnover. Finally, the response to rhTSH administration in bone markers was similar in members of long-lived families as controls, indicating that, at least in bone tissue, offspring and controls respond similarly to this challenge of the thyroid axis. The differences between offspring from long-lived families and controls in maintenance and repair of other tissues, as well as the many variables regarding the specific and direct influence of TSH, fT4 and fT3 on bone turnover markers, remain to be elucidated in further research.

The baseline differences in bone markers between offspring and controls are in line with briefly mentioned findings from a previous subgroup of the Leiden Longevity Study cohort [20] and could be indicative of a generally lower rate of tissue turnover that might contribute to the offspring’s longevity phenotype. Further studies into maintenance and repair of other tissues could provide greater insights whether a lower rate of tissue turnover is a generalized phenomenon in offspring from long-lived families compared to controls, or whether this effect is specific to the bone. It remains subject to speculation whether the difference in thyroid status plays a role in the underlying mechanism (7).

Following rhTSH administration, in this novel and explorative study on the influence of a thyroid axis challenge on markers of bone turnover in a healthy, high middle-aged population, we show that a single dose of 0.1mg rhTSH transiently increased bone turnover.

Previous studies report conflicting findings when it comes to direct effects of TSH on bone turnover, from a positive to a negative to no association, although most studies report a negative association between TSH and bone remodelling [12, 21]. The TSH receptor (TSHR) has been identified in osteoclasts and osteoblasts, but at such low levels that a physiological role of TSH on bone tissue was deemed unlikely [22]. It could be that a challenge situation with rhTSH was able to adequately trigger the TSHR to influence bone tissue despite the low levels of its expression in bone cells [21].

Still, in our study, we are not able to disentangle the influence of TSH from the influence of thyroid hormones on bone turnover markers, since we studied healthy offspring and controls, as opposed to subjects who have undergone a thyroidectomy (in which the direct effect of rhTSH on bone turnover markers could be measured). In participants with an intact thyroid gland, conclusions about TSHR responsivity or direct effects of TSH on bone tissue cannot be drawn because any effect of rhTSH cannot be disentangled from the effect of thyroid hormones.

We do show that rhTSH administration, which is a condition of increasing exogenous circulating TSH and increasing endogenous circulating thyroid hormone levels, is able to transiently increase bone turnover. Moreover, following rhTSH administration, it is mostly levels of thyroid hormone fT4 that are increased [9], and our findings here support the role of thyroid axis, whether through TSH or thyroid hormone, in increasing bone turnover. In future studies, we aim to investigate bone turnover following a challenge with thyroid hormones specifically.

Despite our previous observation of a difference in thyroid responsivity to rhTSH between offspring and controls [9], we did not find a difference in their bone markers’ responses to rhTSH. This could be due to slight increases in thyroid hormone levels already being sufficient in maximally increasing bone markers, therefore even lower thyroidal responsivity to rhTSH in offspring might already be adequate in maximally increasing bone marker concentrations. The influence of thyroid hormones on bone marker concentrations remains our target for future research.

Our study had several strengths. All samples were fasted and obtained in the mornings at 24 h intervals which means that our measurements were minimally disturbed by circadian rhythm of the bone markers or meal variation, known to especially influence CTX to a large degree [20].

This study also has a number of limitations. The main limitation of our study concerns the study design, which was not primarily established for measurement of bone markers. This creates uncertainty regarding the sample size as well as regarding other factors besides the thyroid axis that might influence bone tissue turnover, such as physical stress, steroids, parathyroid hormone, calcitonin and growth hormone (GH) – information on which was not obtained during the rhTSH study and could not be accounted for in our analyses.

Although the TSH challenge study has been powered on the analysis of the primary endpoint, the analyses of secondary endpoints as presented in this paper do represent preconceived explorative analyses. The value of this study as compared to other studies in healthy humans which are observational in design, is that we were able to study the time effect of transient changes in thyroid status in two groups of healthy humans, a group enriched for familial longevity and a control group. In future research, we suggest further investigation in a larger sample of the Leiden Longevity Study of bone phenotypes (using imaging techniques) and including measurements of serum concentrations of calcium, cholecalciferol and metabolites, calcitonin, parathyroid hormone, cytokines and IGF-I, which all impact on bone metabolism. Previously, GH, which also impacts bone metabolism was shown to be different in familial longevity [23, 24].

Another limitation is the existence of uncertainty regarding the selection of familial longevity. By design of the Leiden Longevity Study, the offspring are derived from long-lived families, while controls are the partners of the offspring from long-lived families. Although this design does allow for a comparison between (offspring and control) groups that are matched for age and important adult environmental factors such as socio economic status, a limitation of this design is that contrast between groups may be diluted, as some offspring may be less likely than others to have inherited a favourable genetic predisposition, while some controls may actually also have long-lived parents. Despite this limitation, maternal age and paternal age were higher in offspring compared to controls in accordance with the longevity phenotype selection criteria. To maximise the contrast in familial longevity between groups, future studies should focus on the use of scores which include family longevity history information for both offspring and controls, such as Longevity Relatives Count scores [25].

## METHODS

### Study participants

The LLS was founded in 2002 and designed to investigate genotypes and phenotypes of long-living families (men aged 89 and older, women aged 91 and older) living in The Netherlands in early 2000s [6], without any restrictions on health or demographics [7]. The offspring of these families were asked to participate in the study, with their current partners as controls, thereby creating a case group enriched for longevity and a control group with similar lifestyle factors and socio-economic status, but without selection for familial predisposition to longevity.

Recruitment and study details regarding the study have been described elsewhere [9, 10]. In brief, offspring and controls from the LLS subgroup previously studied in terms of thyroidal status [8] were recruited, with the following exclusion criteria: laboratory results (haemoglobin < 7.1 mmol/L, TSH > 4.0 mU/L , fT4 < 9 pmol/L or > 24 pmol/L, TPO antibody positivity (>35 kU/L)), medical history (cardiac arrhythmias, (history of) thyroid diseases, renal, hepatic or endocrine disease, or any other significant chronic disease), medication use (hormone therapy, thyroid medication), lifestyle factors (nicotine abuse, (history of) alcohol abuse (>28 units per week)) and other factors (difficulty inserting an intravenous cannula, participation in other research projects within the last 3 months, participation in two or more projects in one year, evaluation by a physician as too frail or vulnerable to participate).

### Study protocol

Participants were admitted into the study after passing medical screening. The rhTSH study consisted of four consecutive study days at Leiden University Medical Centre. On the morning of study day 1, an intravenous cannula was placed in a forearm vein, blood was withdrawn at baseline and rhTSH was administered through intramuscular injection (recombinant human TSH, 0.1 mg/mL in 1 mL, gluteal muscle). The time of injection was used as reference, time zero. On study day 2, 3 and 4, fasted blood samples were obtained at respectively 24, 48 and 72 h after rhTSH injection.

During study day 1, subjects were mostly sedentary in the study room and received standardized meals (Nutridrink Compact, Nutricia Advanced Medical Nutrition, Zoetermeer, The Netherlands). During other study days, subjects visited the LUMC in the morning and were at their leisure the rest of the day; meals were not standardized.

Height, weight and body composition were measured on day 2. Body composition was measured with a Bioelectrical Impedance Analysis meter at a fixed frequency of 50kHz (Bodystat 1500 Ltd, Isle of Man, British Isles [26]). Exercise level was determined by a questionnaire about type of exercise, duration and weekly frequency.

The studies were designed in accordance with the declaration of Helsinki and have been approved by the Medical Ethical Committee of the Leiden University Medical Centre. They are registered at Leiden University Medical Centre under the protocol P16.107 and with EudraCT under the number 2016-001497-15. All subjects gave written informed consent prior to medical screening.

### Handling of samples

Serum samples were kept at room temperature for 60 min to clot before processing at the Department of Clinical Chemistry and Laboratory Medicine, Leiden University Medical Centre, The Netherlands. Samples were centrifuged for 10 min at 2350 G relative centrifugal force at a temperature of 20°C. After being transferred to 500 microliter aliquots, serum samples were temporarily stored at –20°C prior to permanent storage at –80°C until analysis.

### Laboratory measurements

Laboratory measurements in serum samples were performed after all subjects had completed the study. All measurements were performed with the same lot number. For each participant, samples from the different time points were measured in the same batch.

### Assays and assay performance

All measurements were performed with fully automated, software monitored equipment and diagnostics from Roche Diagnostics (Almere, The Netherlands) at the Department of Clinical Chemistry and Laboratory Medicine at Leiden University Medical Centre, The Netherlands. Aspartate aminotransferase (AST) (Catalogue number 11876848216), alanine aminotransferase (ALT) (Catalogue number 11876805216) and creatine (Catalogue number 5168589190) for estimating glomerular filtration rate (GFR) were measured from a fasted morning serum sample using the Modular P800 clinical chemistry analyser. GFR was calculated using the CKD-EPI calculation [27]. Thyroid parameters TSH (Catalogue number 11731459122, research reference identifier (RRID): AB_2756377), fT4 (Catalogue number 6437281190, RRID: AB_2801661), T4 (Catalogue number 12017709122, RRID: AB_2756378), fT3 (Catalogue number 6437206190, RRID: AB_2827368) and T3 (Catalogue number 11731360122, RRID: AB_2827369), and bone markers (CTX and P1NP) were measured in serum by an immunoassay using Roche cobas8000 with an E602 module. The coefficients of variation (CV) were 2.33 (SD 0.01) for CTX, 2.28 (SD 0.77) for P1NP, 2.36 (SD 0.52) for TSH and 5.55 (SD 2.28) for fT4.

### Statistical analysis

Descriptive statistics were used to summarise group characteristics. Independent samples *T* test, Mann-Whitney *U* test and Chi square test were used, depending on the characteristics of the variable (normally distributed, not normally distributed and categorical, respectively), to statistically test for differences between offspring and controls regarding baseline characteristics. General linear modelling (GLM) was used for repeat measurements (analysis of variance, ANOVA) to investigate the effects of time and sex on the concentration profiles of CTX and P1NP, and the interaction term time^*^ (offspring/control) status was used to test for differences in time dependent changes in CTX and P1NP between offspring and controls. In all analyses, *P* ≤ 0.05 was considered statistically significant.

Programs used for statistical analyses were SPPS for Windows, version 23 (SPSS, Chicago, IL), Systat version 13 (Systat Software, Inc, San Jose, CA) and Matlab (The MathWorks Inc, Natick, MA). Graphs were made using Microsoft Office Excel 2016 and GraphPad Prism for Windows, version 8.1.1 (330) (GraphPad Software, Inc, San Diego, CA).
